# Acute obstructive pyelonephritis due to pyosalpinx: a case report

**DOI:** 10.1186/s13256-023-03900-6

**Published:** 2023-04-25

**Authors:** Kays Chaker, Yassine Ouanes, Eya Azouz, Mohamed Trigui, Anouar Madani, Boutheina Mosbahi, Wiem Elabed, Oumayma Chbeb, Mokhtar Bibi, Kheireddine Mrad Dali, Adel Ammous, Yassine Nouira

**Affiliations:** 1 Department of Urology, La Rabta Hospital, University of TUNIS El Manar, Tunis, Tunisia; 2 Department of Radiology, La Rabta Hospital, University of Tunis El Manar, Tunis, Tunisia; 3 Department of Anesthesia, La Rabta Hospital, University of Tunis El Manar, Tunis, Tunisia

**Keywords:** Pelvic inflammatory disease, Pyosalpinx, Pyelonephritis, Drainage

## Abstract

**Background:**

A pyosalpinx is the acute inflammation of the fallopian tube, which fills up and swells with pus. It commonly results from inadequate or delayed treatment of pelvic inflammatory disease.

**Case presentation:**

We report the case of a 54-year-old Africain female patient, who presented with sustained high-grade fever, right flank pain, and severe acute storage low-urinary-tract symptoms. Computed tomography showed signs of acute obstructive pyelonephritis with a right tubular juxtauterine mass with complex internal fluid and thick enhancing walls exerting a mass effect on the right ureter. A drainage of the right excretory cavities by a JJ stent was performed. An ultrasound-guided aspiration of the collection was also performed.

**Conclusion:**

A pyosalpinx can then exert a mass effect on the excretory cavities, thus causing an acute obstructive pyelonephritis. A double drainage coupled with an effective antibiotic therapy is then necessary.

## Background

Pyosalpinx results when the infected fallopian tube is occluded at its fimbrial end, and the distal part of the tube becomes distended with the collection of pus [1]. It is a very rare gynecological pathology during adolescence and even rarer in sexually inactive females [1, 2].

## Case presentation

**Fig. 1:**
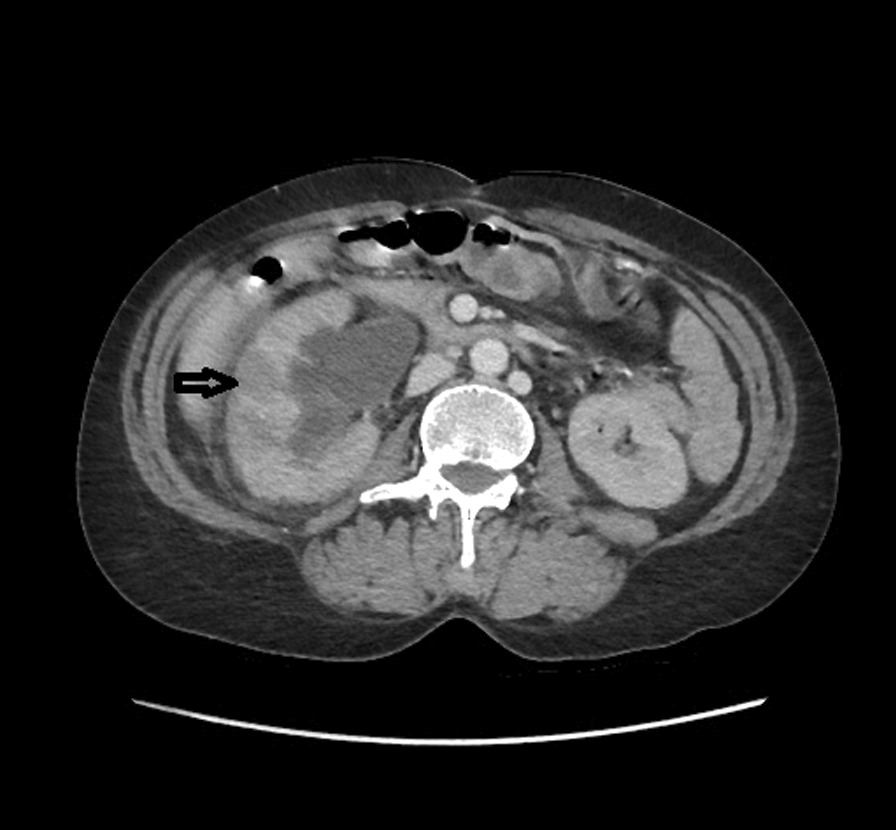
Computed Tomography showed a right hydroureteronephrosis, infiltration of the right peri-renal fat, multiple nephritis foci (Arrow)

**Fig. 2:**
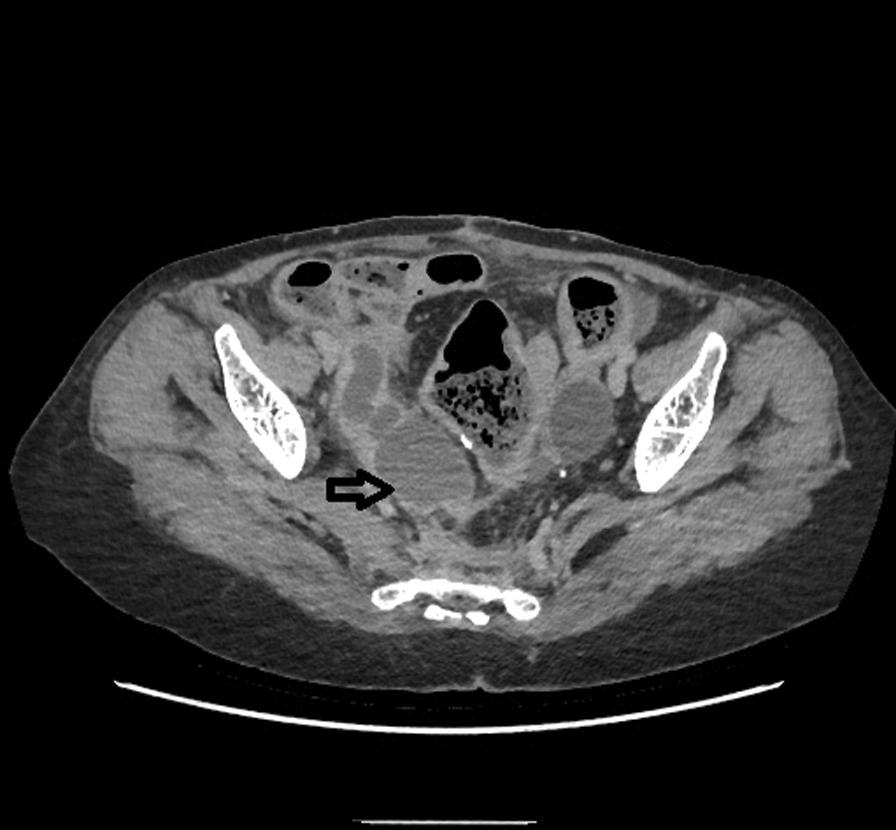
Computed Tomography findings include a right tubular juxta-uterine mass with complex internal fluid and thick enhancing walls (Arrow)

**Fig. 3:**
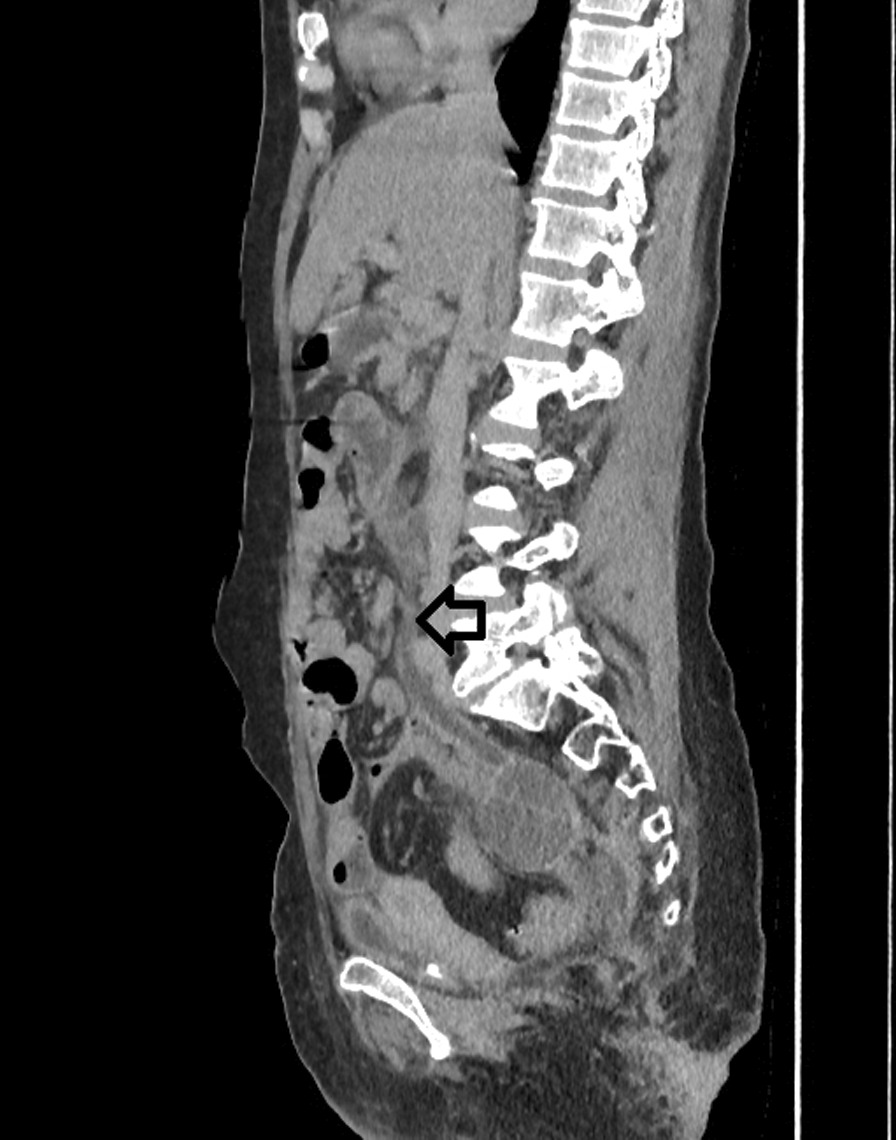
Scannographic aspect of a pyosalpinx (Arrow) exerting a mass effect on the right ureter responsible for a right obstructive renal syndrome

**Fig. 4:**
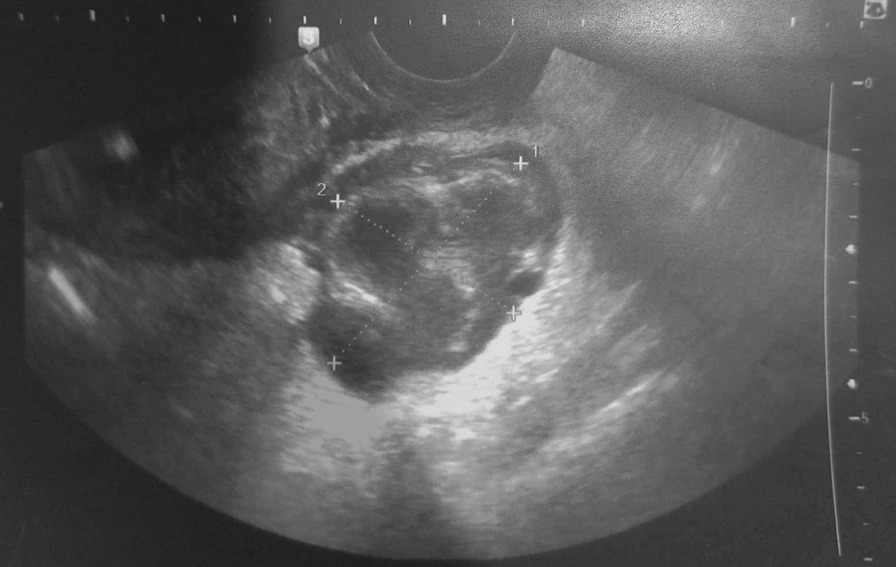
Ultrasound showing an irregular adnexal cystic mass with a thin wall, measuring 6 cm. The “cogwheel sign” suggested that mass was a pyosalpinx

A 56-year-old menopausal multiparous Africain female with well-controlled hypertension and type 2 diabetes mellitus, presented to the emergency department for sustained high-grade fever, right flank pain, and severe low-urinary-tract symptoms (LUTS). On physical examination, she was in mild distress due to pain. Triage vitals showed a blood pressure of 100/56 mmHg, a heart rate of 120 beats per minute, a temperature of 39.4 °C, a respiratory rate of 22 breaths per minute, and an oxygen saturation status of 95% in room air. An abdominal examination revealed a soft abdomen with mild diffuse generalized abdominal tenderness and right renal angle tenderness; however, no guarding or rigidity was observed. All other physical examinations, including the central nervous system examination, were normal. Blood work revealed a white blood cell count of 18.7 × 10^3^/dl, a mild anemia, and a creatinine level of 7 mg/l; C-reactive protein (CRP) level was 294.6 mg/l. Urinalysis showed combined hematuria and pyuria with the urine culture demonstrating the growth of > 10^5^ *E. coli*. Then patient underwent a contrast-enhanced computed tomography (CT) of the abdomen and pelvis. CT showed a right hydroureteronephrosis, infiltration of the right perirenal fat, and multiple nephritis foci (Fig. 1). In addition, CT findings include a right tubular juxtauterine mass with complex internal fluid and thick enhancing walls (Fig. 2), exerting a mass effect on the right ureter (Fig. 3). Ultrasound showed an irregular adnexal cystic mass with a thickened wall, measuring 6 cm. The “cogwheel sign” suggested that the mass was a pyosalpinx (Fig. 4). Testing for *Chlamydia trachomatis*, *Neisseria gonorrhoeae*, reactive plasmin reagin, and human immunodeficiency virus was negative. She was started on a combination of ceftriaxone, metronidazole, and doxycycline along with paracetamol. A ureteric stent was placed. An ultrasound probe was placed transvaginally. An 18-gauge needle was advanced into the collection, and 50 ml of fluid was aspirated. The material was partially complex and partially simple in character. The fluid collection collapsed entirely, and the needle was removed. The culture was polymicrobic. It was negative for *Chlamydia trachomatis* and *Neisseria gonorrhoeae*. Tuberculosis was also ruled out on further workup. She was discharged after 3 days, on oral amoxicillin for 10 days. At 2 month follow-up, she was fine and asymptomatic.

## Discussion

Suspicion of upper genital infection is a frequent clinical situation in gynecological emergencies and in primary care consultations. Pyosalpinx and tubo-ovarian abscess are almost always complications of pelvic inflammatory disease and are sexually transmitted infections in several cases. Pyosalpinx is defined as purulent intratubal collections, which may occur *de novo* or by ascending infection of a hydrosalpinx. Therefore, pyosalpinx and tubo-ovarian abscess are usually observed in young women; they are rarely found in older women [3]. In the literature, 194 cases of pyosalpinx or tubo-ovarian abscess in postmenopausal woman have been reported [3, 4]. Pyosalpinx may present with very few specific symptoms or remain silent [5]. Less than 50% of women with pyosalpinx present with fever and chills [5, 6]. Other symptoms include nausea, vaginal discharge, and abnormal vaginal bleeding [5, 6]. Functional urinary signs are reported in 15–30% of upper genital infections [7]. On physical examination, patients may show tenderness over the adnexal region with or without guarding or rebound. The absence of specific symptoms and conclusive signs during the physical examination may delay a proper diagnosis [5]. In the majority of cases, salpingitis results from a sexually transmitted ascending infection [2]. In sexually inactive females, biological factors may play a role in the development of infection, including decreased level of protective antibodies, relative larger zone of cervical ectopy, greater permeability of cervical mucus, and alteration of vagina flora [2]. The initial imaging modality of choice for the diagnosis of pyosalpinx is transvaginal ultrasound, because it is cost-effective and allows detailed visualization of pelvic structures. Ultrasound can show a dilated serpentine/tubular structure in the pelvis. Low-level echoes due to the higher protein content of the debris within the tube distinguish a pyosalpinx from a hydrosalpinx. Abdominopelvic computed tomography with contrast injection is often performed in an emergency setting. Typically, the pyosalpinx forms an elongated pseudocystic image, latero-uterine and then curving backwards from the uterus towards the cul-de-sac of Douglas, marked by one or more flexion folds. The wall and folds appear thick and echogenic. The 3D mode allows a more precise analysis of the shape and partitions. Under the probe, particularly vaginally, the mass is fixed and painful. Magnetic resonance imaging is a very useful method to examine and diagnose gynecological organs in elderly as well as young women. Magnetic resonance imaging images in the pelvis show a markedly dilated fallopian tube posterior to the ovary, edema surrounding the fallopian tube, and a thickened and enhanced tube wall with active inflammation. The early diagnosis of this pathology is hampered by its rarity and overlapping of symptoms with other causes of the acute abdomen, such as acute appendicitis, cystitis, gastroenteritis, pyelonephritis, and peritonitis [1, 2]. As a therapy for pyosalpinx, it is recommended that antibiotics therapy is started as soon as possible for patients [4, 8]. Pyosalpinx requires prompt diagnosis, admission, intravenous antibiotics, and possibly aspiration or surgery [5, 9]. Treatment of pyosalpinx varies from conservative management with intravenous antibiotics to laparoscopic aspiration, image-guided aspiration or drainage, laparoscopic salpingostomy, or salpingectomy [5, 10]. In more than 75% of patients, antibiotics alone may be sufficient for treating pyosalpinx [11]. In case of a collection > 3–4 cm, drainage should be performed because the failure rate is higher in the absence of drainage as is the risk of serious complications [12].

## Conclusion

Pyosalpinx is a less severe form of pelvic inflammatory disease that leads to shorter hospital stays and more favorable outcomes than tubo-ovarian abscess. Pyosalpinx unrelated to sexually transmitted infection is rare but should be considered in non-sexually active women. Imaging modalities such as ultrasound and computed tomography can aid in making the diagnosis of pyosalpinx. Early pyosalpinx can be treated successfully with adequate antibiotic coverage. Pyosalpinx when associated with an adjacent abscess usually requires percutaneous or surgical intervention.

## Data Availability

The datasets are available from the corresponding author on reasonable request.
